# Impact of maternal psoriasis on adverse maternal and neonatal outcomes: a systematic review and meta-analysis

**DOI:** 10.1186/s12884-023-06006-5

**Published:** 2023-09-30

**Authors:** Shoboo Rahmati, Hossein Moameri, Neda Malek Mohammadi, Mojtaba Norouzi, Nima Ghalekhani, Amin Beigzadeh, Nasrin Changizi, Hamid Sharifi

**Affiliations:** 1 Department of Epidemiology and Biostatistics, Faculty of Public Health, Kerman University Of Medical Sciences, Kerman, Iran; 2 Reproductive Health Research Center, Clinical Research Institute, Urmia University of Medical Sciences, Urmia, Iran; 3 HIV/STI Surveillance Research Center, and WHO Collaborating Center for HIV Surveillance, Institute for Futures Studies in Health, Kerman University of Medical Sciences, Kerman, Iran; 4 Education Development Center, Sirjan School of Medical Sciences, Sirjan, Iran; 5 Maternal, Fetal and Neonatal Research Center, Tehran University of Medical Sciences, Tehran, Iran

**Keywords:** Psoriasis, Psoriatic arthritis, Maternal outcomes, Neonatal outcomes, Meta-analysis

## Abstract

**Background:**

There is a dearth of robust evidence regarding the correlation between psoriasis with maternal and neonatal outcomes, making it challenging to establish definitive recommendations for the management of these patients. This systematic review and meta-analysis aimed to review the evidence with regard to the impact of maternal psoriasis on maternal and neonatal outcomes.

**Methods:**

Following the PRISMA guideline, a systematic search of English articles using PubMed, Embase, Scopus, ScienceDirect, Web of Science, Google Scholar, and the Cochrane Library was conducted. The search was performed from inception to 22^nd^ of May 2022.

**Result:**

A significant association was observed between psoriasis and maternal outcomes, including cesarean delivery [OR = 1.25 (95% CI: 1.13–1.30, *p*-value = 0.001)], (pre)eclampsia [OR = 1.29 (95% CI: 1.15–1.44, *p*-value = 0.0001)], gestational diabetes [Odds Ratio (OR) = 1.23 (95% Confidence Intervals (CI): 1.15–1.30, *p*-value = 0.001)], gestational hypertension [OR = 1.31 (95% CI: 1.18–1.45, *p*-value = 0.001)] and preterm birth [OR = 1.22 (95% CI: 1.10–1.35, *p*-value = 0.001)]. Also, there was a significant association between psoriasis and neonatal outcomes, including small for gestational age [OR = 1.07 (95% CI: 1.02–1.11, *p*-value = 0.053)], low birth weight [OR = 1.19 (95% CI: 1.02–1.38, *p*-value = 0.001)] and stillbirth [OR = 1.27 (95% CI: 1.04–1.55, *p*-value = 0.023)].

**Conclusion:**

Maternal psoriasis could negatively impact maternal and neonatal outcomes. Our results strengthen the importance of close monitoring of the mothers’ psoriasis status before and during pregnancy.

**Supplementary Information:**

The online version contains supplementary material available at 10.1186/s12884-023-06006-5.

## Introduction

Psoriasis is a severe, non-communicable, and disabling disease that poses a substantial public health concern due to psychological, social, and financial burden. To inform policymakers and healthcare professionals about the impact of this disease on public health, a better understanding of the global burden of psoriasis is required [1]. Psoriasis is caused by the interaction of several factors, including genetics and environmental factors [2]. Although succinct studies on the prevalence of psoriasis are published, it is more likely to occur among white populations and people living at higher latitudes. Studies that report information on the incidence of psoriasis are limited. Despite the fact that the pathophysiology of psoriasis is complex and not fully understood [3], it has been disclosed that type 17 (Th17) helper cells play an important role in pathogenesis and are also associated with adverse pregnancy outcomes [4].

The reported prevalence of psoriasis is substantially varied in different studies. Earlier estimates of the prevalence of psoriasis in adults range between 0.27% [5], and 11.4% [6] with age, sex, geography, ethnicity, genetic, and environmental factors contributing to the variation in the prevalence of the disease [7, 8]. The incidence rate of psoriasis is the highest among the 15–39 and 50–59 age groups [9]. Therefore, women of reproductive age may often be at a higher risk of the disease. Treatment of psoriasis is long-lasting and sometimes needs systemic therapies [10]. In addition, people with psoriasis may have a significantly lower quality of life and greater psychological disorders, including anxiety, sadness, and suicidal ideations [11]. In addition, psoriasis encompasses a broad clinical range of different skin manifestations, such as fistulations, palmoplantar hyperkeratosis, pustular lesions, plaques, and erythema to-squamous papules. It should be noted that just nearly half of the women with psoriasis have the chance of experiencing clinical remission [12].

The risk of adverse pregnancy outcomes may increase due to the spread of psoriasis inflammation and psoriasis-related comorbidities such as diabetes, cardiovascular diseases, and depression [13, 14]. Pregnant women with psoriasis are more likely than healthy pregnant women to experience unfavorable pregnancy outcomes both at the maternal and neonatal levels such as preterm birth, (pre)eclampsia, gestational diabetes, congenital malformations, stillbirth, and low birth weight (LBW) [15]. Inflammation has been linked to unfavorable pregnancy outcomes, including preterm birth and low birth weight. Elevated levels of proinflammatory cytokines associated with active psoriasis, such as IL-6, C-reactive protein, and tumor necrosis factor-α, have been observed in either the mother's serum or cord blood during pregnancies that lead to preterm birth or small-for-gestational-age neonates [16, 17]. Furthermore, other inflammatory conditions characterized by similar immune-mediated mechanisms, such as inflammatory bowel disease and rheumatoid arthritis, have also been linked to an increased risk of preterm birth and low birth weight [18, 19]. Based on the available data, there is conflicting evidence regarding pregnancy outcomes in women with psoriasis [12]. Some studies have indicated an elevated risk of adverse pregnancy outcomes among women with psoriasis, whereas others have not proved this association [12, 13]. Consequently, due to the inconsistency of these findings, it remains unclear whether psoriasis has an impact on pregnancy outcomes. In this regard, we conducted a systematic review and meta-analysis of observational studies to shed light on these conflicting results and evaluate the impact of psoriasis on maternal and neonatal outcomes.

## Method

### Study design

This study was undertaken according to the PRISMA (Preferred Reporting Items for Systematic Reviews and Meta-Analyses) guideline [20]. These steps were taken into consideration: 1) developing a data sources and search strategy, 2) inclusion and exclusion criteria, 3) study selection, 4) Risk of bias assessment, 5) data extraction, 6) data analysis, and 7) sensitivity analysis including measuring heterogeneity and publication bias as well as the measurement of association.

#### Data sources and search strategy

A pre-designed search strategy was employed to thoroughly explore published English literature in electronic databases, namely PubMed, Embase, Scopus, ScienceDirect, Web of Science, Google Scholar, and the Cochrane Library. No time limit was considered for article searching and all searches were conducted until May 22, 2022. Keywords used included psoriasis, psoriatic arthritis, pregnancy, pregnant, birth, cohort, and case–control studies. The reference lists of selected articles were also searched to identify additional potentially relevant articles as well. The detailed search strategy of PubMed database is depicted in Additional file 1. Two reviewers worked independently on each step. A third reviewer resolved discrepancies between the results.

#### Inclusion and exclusion criteria

All observational studies which reported the impact of psoriasis or psoriatic arthritis in pregnant women and maternal outcomes (e.g., spontaneous abortion, cesarean delivery, preterm birth (defined as babies born alive before 37 weeks of pregnancy are completed), (pre)eclampsia, gestational hypertension, and gestational diabetes or neonatal outcomes (e.g., low birth weight (defined as birth weight < 2.5 kg), small for gestational age (a newborn with a bodyweight less than 10% for gestational age), stillbirth (defined as intrauterine death before or during Childbirth), congenital malformations, Apgar score < 7, and premature rupture of membranes were included. We excluded articles that did not contain original data, such as reviews, commentaries, literature reviews, and case studies. Additionally, unpublished articles, non-peer-reviewed articles, and qualitative studies were also excluded from the analysis.

#### Study selection

The studies were chosen in two stages: a preliminary screening of the title and abstract, followed by a full-text review of potentially eligible articles. Two reviewers (MN and HM) independently screened the retrieved studies. Any disagreements between investigators were resolved through discussion and the decision of a third investigator (SR). The Kappa statistics was used to assess the two investigators' agreement and inter-reliability. The kappa coefficient was 80%.

#### Risk of bias assessment

STROBE (Strengthening the Reporting of Observational Studies in Epidemiology) checklist was used to assess the risk of bias in included studies [21]. A score of 0–22 was allocated to each study. The final score of ≥ 16 was considered high quality.

#### Data extraction

To minimize data collection errors, a standardized data abstraction form was employed. The form included fields such as author name, publication year, country, study design, data source, study setting, enrollment period, specific details on psoriasis or psoriatic arthritis, demographic and clinical characteristics of the disease, comparative data, and the relevant outcomes of interest. In cases of ambiguities in a particular study, attempts were made to resolve them by contacting the authors through email.

#### Data analysis

To assess heterogeneity in the study outcomes, I^2^ statistics were used to report the heterogeneity across studies in three categories, including low heterogeneity ≤ 25%, moderate heterogeneity between 25 to 75%, and high heterogeneity ≥ 75% [22]. Subgroup analyzes were conducted based on the continent type and study quality. The visual funnel plot and the Egger test measure of association were used to assess publication bias [23, 24]. A random-effect meta-analysis, using *db metan* command in Stata 14.2 (Stata Corp, College Station, TX, USA) with 95% confidence intervals (CI), was used to calculate the pooled measure of association, and the pooled relative risk (RR) or odds ratio (OR). As some studies reported OR and some others reported RR as the measure of the effect, and due to the variation of baseline risks in the articles, we could not change these two measures; so, we analyzed data in two subgroups based on the reported RR or OR. As the RR has been considered as a measure of association in a number of studies, we presented the output of RR in Additional file 2.

#### Sensitivity analysis

A sensitivity analysis was conducted to determine the robustness of the outputs. In each stage, one study was dropped, and the results were compared to the full analysis.

## Result

### Overview of search

**Fig. 1:**
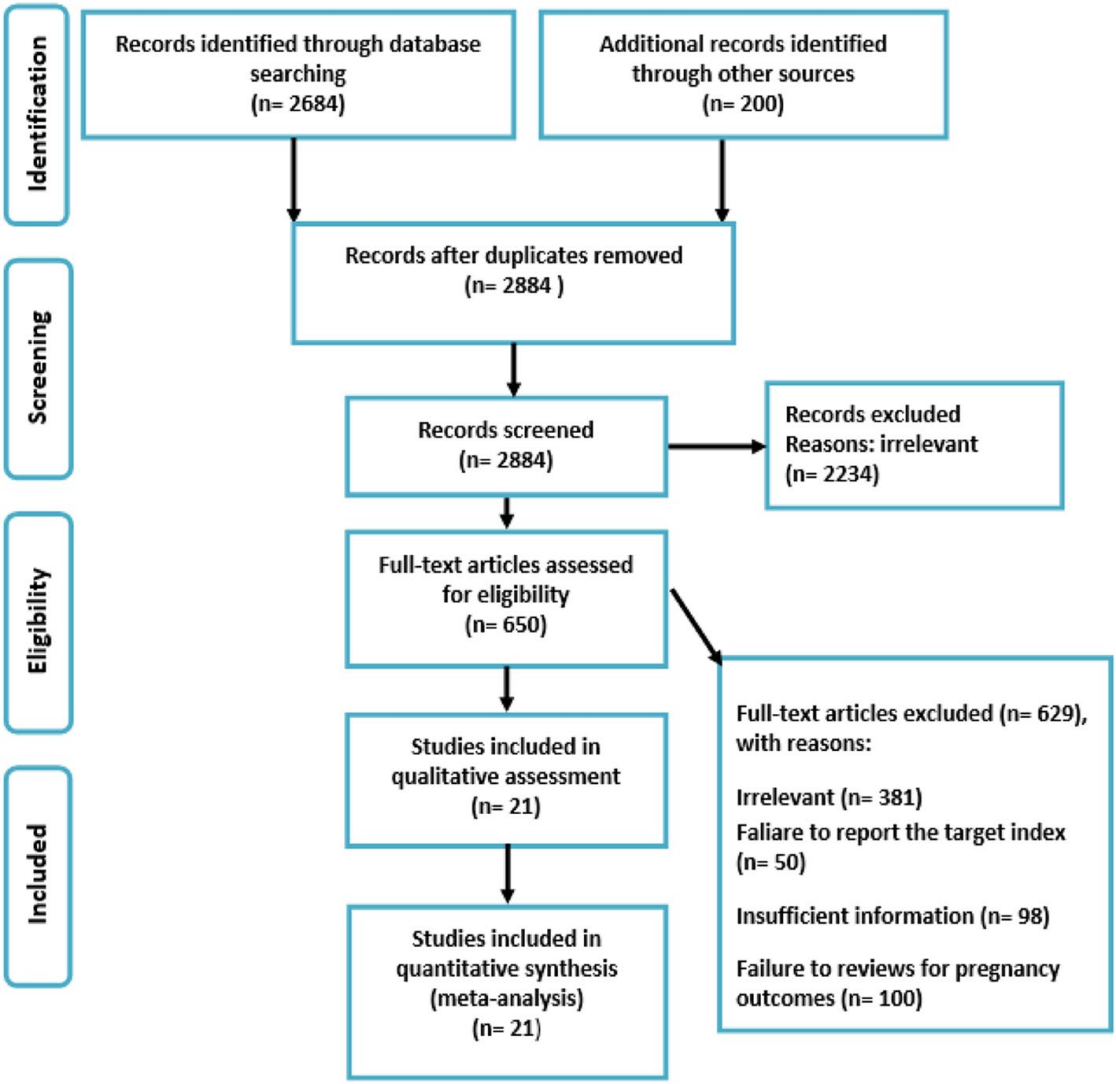
A flow chart depicting the stages of retrieving articles and checking eligibility criteria for meta-analysis to assess the impact of psoriasis on pregnancy and neonatal outcomes

**Fig. 2:**
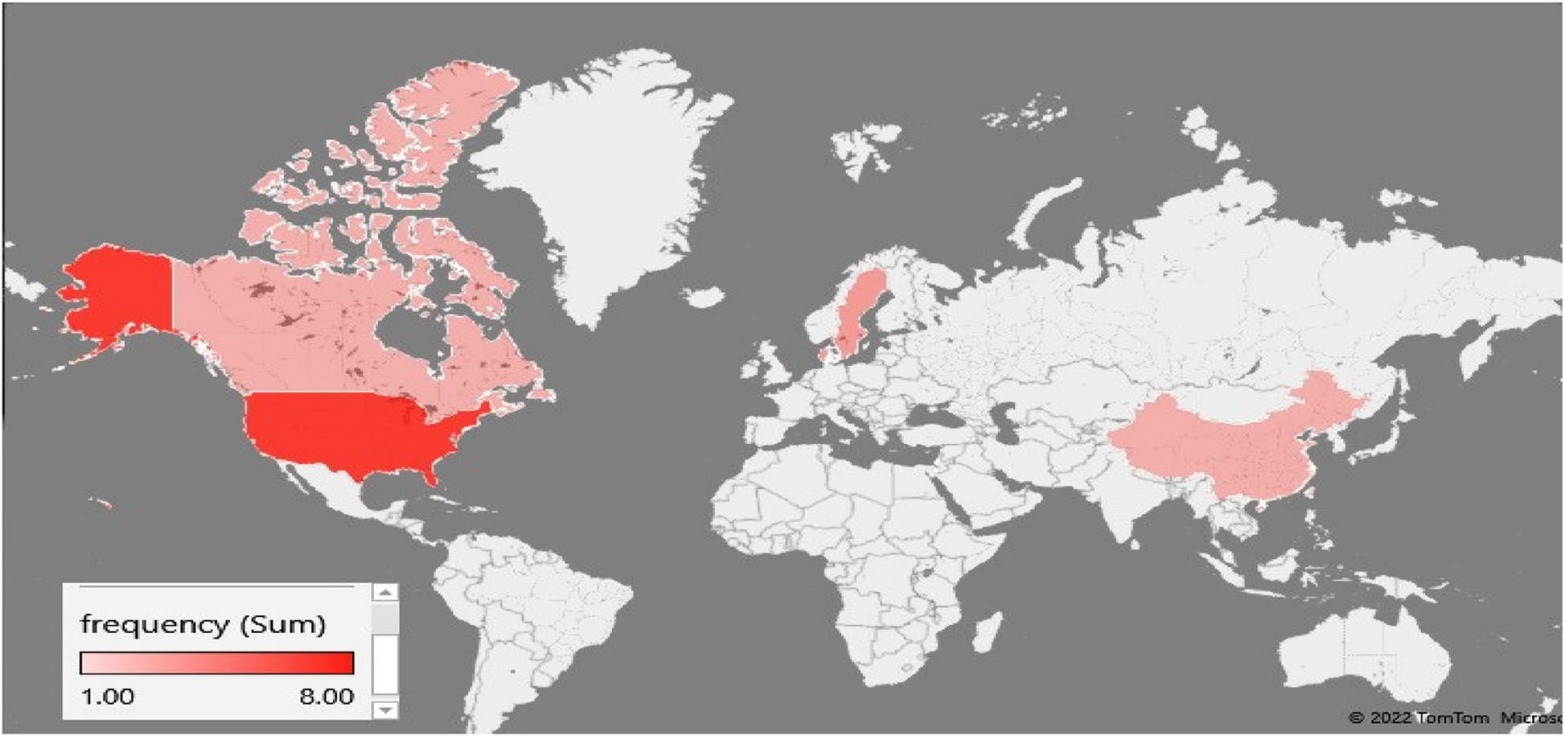
Geographical distribution of included studies on the impact of psoriasis on maternal and neonatal outcomes

In the primary search in different databases, 2,884 studies were identified. After the title, abstract and full-text screening, 21 studies were included for analysis. The included studies consisted of 36,911,079 pregnant women, of which 82,662 women with psoriasis and 36,828,417 controls (Fig. 1 and Table 1). A descriptive summary of included studies concerning study location and the setting is shown in Fig. 2. The most included studies were in the United States (8 articles; 38.0%). After that, Denmark and Sweden (3 articles for each country; 14.3%). The geographical distribution of the included studies is presented in Fig. 2.

**Table 1:** Characteristics of included studies on the impact of psoriasis on maternal and neonatal outcomes

Reference	Author	Publication year	Study type	N psoriasis	study period	Continent	Country	Quality score	Quality
[25]	Seeger GD, 2007	2007	Prospective	4262	1997–2002	America	USA	20	High
[26]	Ben-Dav, 2008	2008	Retrospective	84	1988–2004	Asia	Israel	14	Low
[27]	Cohen-Barak E, 2010	2010	Retrospective	35	1999–2009	Asia	Israel	15	Low
[28]	Lima XT, 2011	2011	Retrospective	122	1999–2009	America	USA	18	High
[29]	Yang YW, 2011	2011	Retrospective	1463		Asia	China	14	Low
[30]	Harder E, 2014	2014	Prospective	2553	1996–2003	Europe	Denmark	10	Low
[31]	Amiri, 2016	2016	Prospective	180	2005–2015	America	USA	16	High
[32]	Bandoli G, 2017	2017	Prospective	330	2009–2015	America	USA	17	High
[33]	Chiou MJ, 2017	2017	Retrospective	3669	2001–2012	Asia	China	18	High
[34]	Broms G, 2018	2018	Prospective	8097	2007–2012	Europe	Denmark	16	High
[35]	Polachek A, 2018	2018	Prospective	74		America	Canada	16	High
[36]	Remaeus K, 2019	2019	Retrospective	541	1997–2014	Europe	Sweden	18	High
[37]	Smith CF, 2019	2019	Prospective	117	2004–2018	America	USA	15	Low
[38]	Strouse J, 2019	2019	Retrospective	10,975	2007–2012	America	USA	18	High
[39]	Bandoli G, 2020	2020	Retrospective	1255	2007–2012	America	USA	10	Low
[40]	Lambe M, 2020	2020	Retrospective	33,488	1992–2009	Europe	Sweden	11	Low
[41]	Huang YH, 2020	2020	Retrospective	4058	2001–2012	Asia	Taiwan	15	Low
[42]	Remaeus K, 2022	2022	Retrospective	921	2007–2017	Europe	Swedish	20	High
[43]	Gangbe EM, 2021	2021	Retrospective	419		America	Canada	17	High
[44]	Johansen CB, 2022	2022	Retrospective	6282	2004–2017	Europe	Denmark	21	High
[45]	Krim D, 2021	2021	Retrospective	3737	1999–2015	America	USA	20	High

### Effect size based on OR

#### The impact of psoriasis on maternal outcomes

**Fig. 3:**
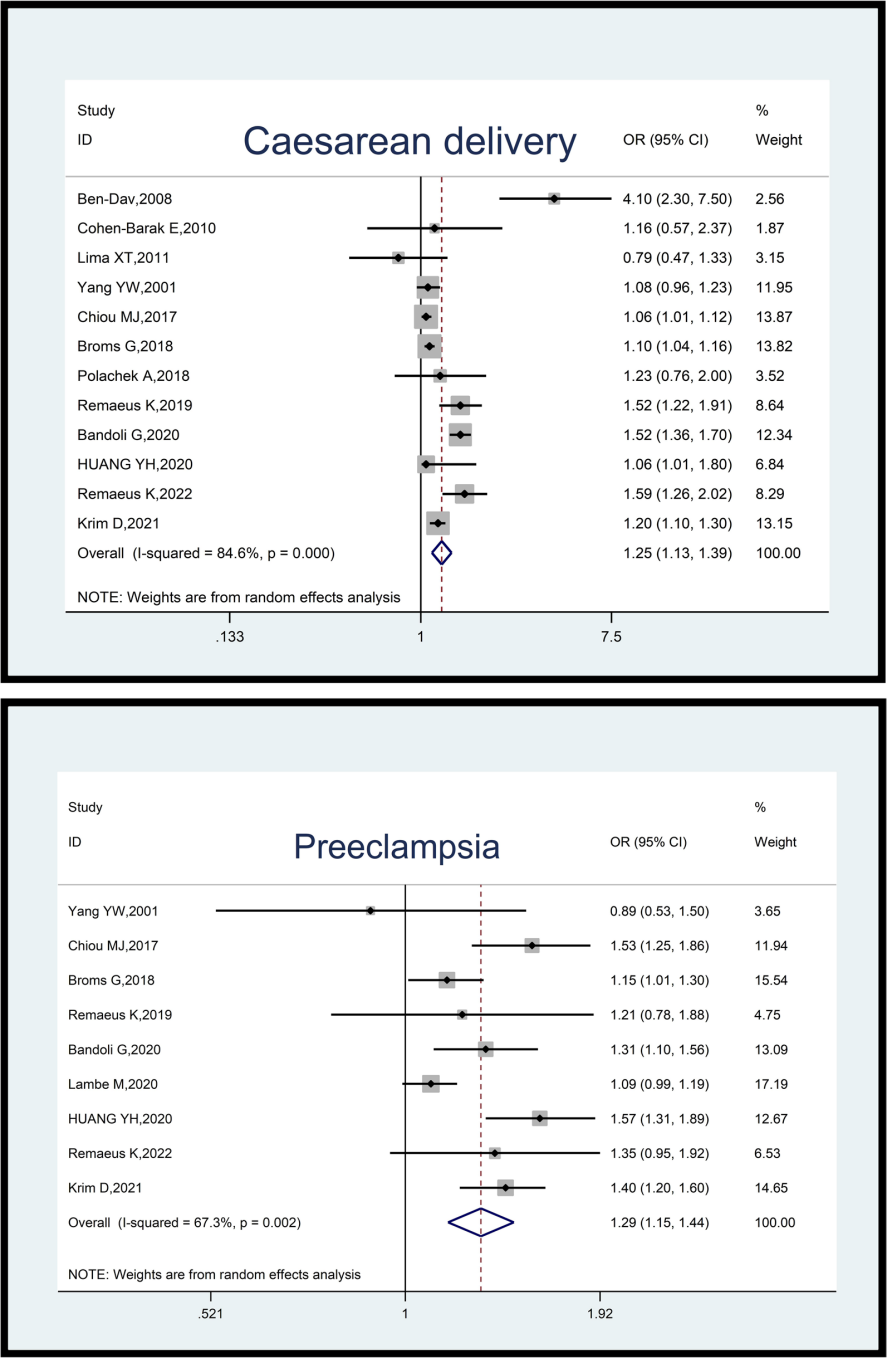
Impact of psoriasis on maternal outcomes (Caesarean delivery and Preeclampsia)

A meta-analysis of 12 studies involving a total sample size of 22,463,619 pregnant women (24,056 cases and 22,439,563 controls) revealed that psoriasis was associated with an increased odds of cesarean delivery (OR; 1.25, 95% CI: 1.13–1.39, *p*-value = 0.001). The heterogeneity among included studies was substantial (I^2^ = 84.6%; *p*-value < 0.001) (Fig. 3).

In the analysis of nine studies comprising 24,135,106 pregnant women (66,145 cases and 24,068,961 controls), it was found that psoriasis was linked to an increased odds of (pre)eclampsia (OR: 1.29, 95% CI: 1.15–1.44, *p*-value = 0.001). The included studies demonstrated moderate heterogeneity (I^2^ = 67.3%; *p*-value = 0.002) (Fig. 3).

**Fig. 4:**
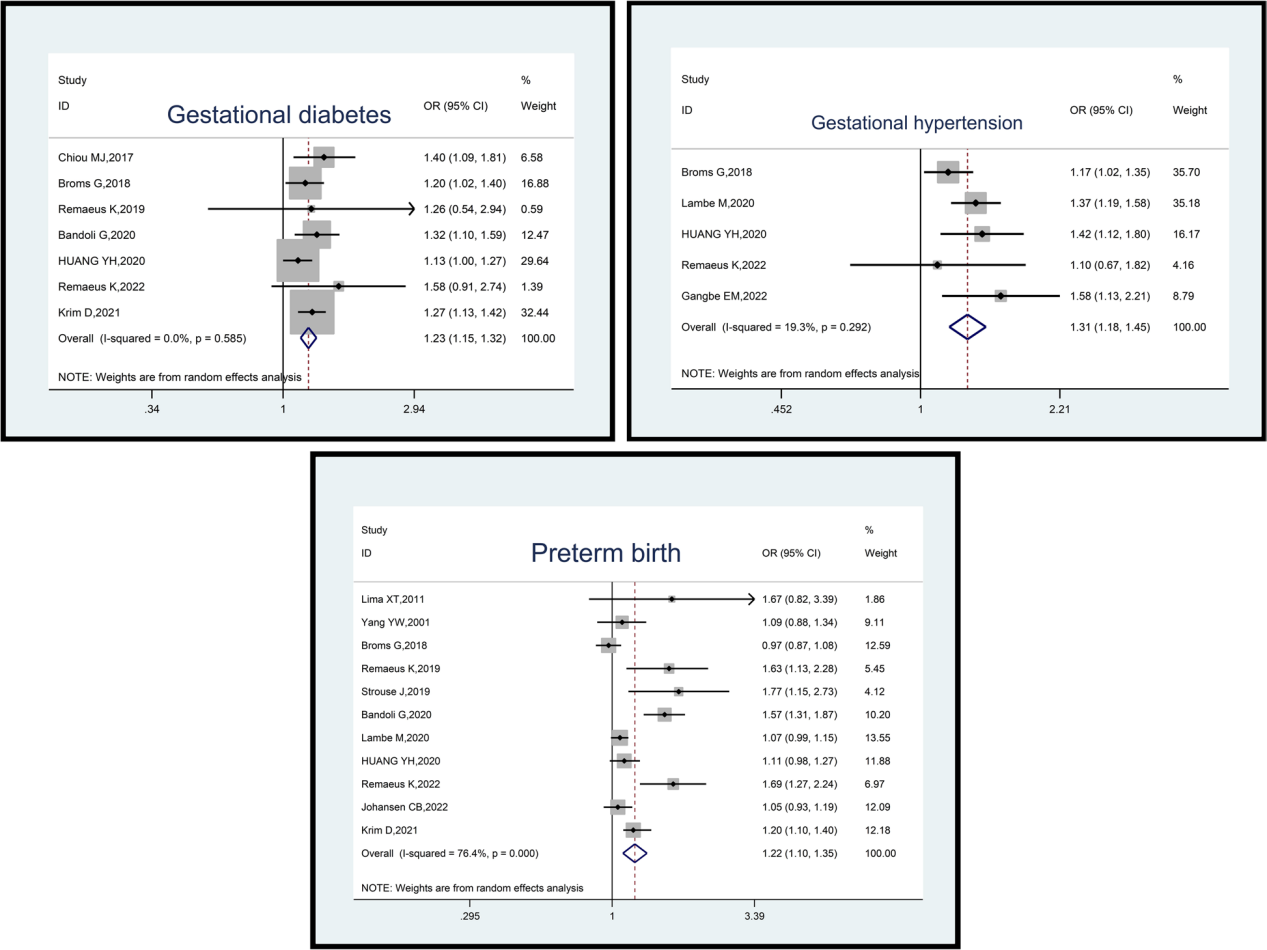
Impact of psoriasis on maternal outcomes (Gestational diabetes, Gestational hypertension and Preterm birth)

Among the seven studies involving 22,461,633 pregnant women (22,278 cases and 22,439,355 controls), psoriasis was associated with an elevated odds of gestational diabetes (OR: 1.23, 95% CI: 1.15–1.32, *p*-value = 0.001). The included studies exhibited low heterogeneity (I^2^ = 0.0%; *p*-value = 0.585) (Fig. 4).

Examining five studies encompassing 14,077,329 pregnant women (46,983 cases and 14,030,346 controls), it was found that psoriasis increased the odds of gestational hypertension (OR: 1.31, 95% CI: 1.18–1.45, *p*-value = 0.001). The heterogeneity among these studies was low (I2 = 19.3%; *p*-value = 0.292) (Fig. 4).

The analysis of 11 studies involving 24,940,770 pregnant women (70,939 cases and 24,869,831 controls), findings revealed that psoriasis was positively associated with an increased odds of preterm birth (OR: 1.22, 95% CI: 1.10–1.35, *p*-value = 0.001). The included studies exhibited high heterogeneity (I^2^ = 76.4%; *p*-value = 0.001) (Fig. 4).

Analysis of three studies, with a sample size of 378 pregnant women (231 cases and 147 controls), showed that psoriasis had no significant association with spontaneous abortion OR = 1.83 (95% CI: 0.85–3.95, *p*-value = 0.1). Heterogeneity among the included studies (I^2^ = 81.7%; *p*-value = 0.004) showed a high heterogeneity value and it was significant.

#### The impact of psoriasis on neonatal outcomes

**Fig. 5:**
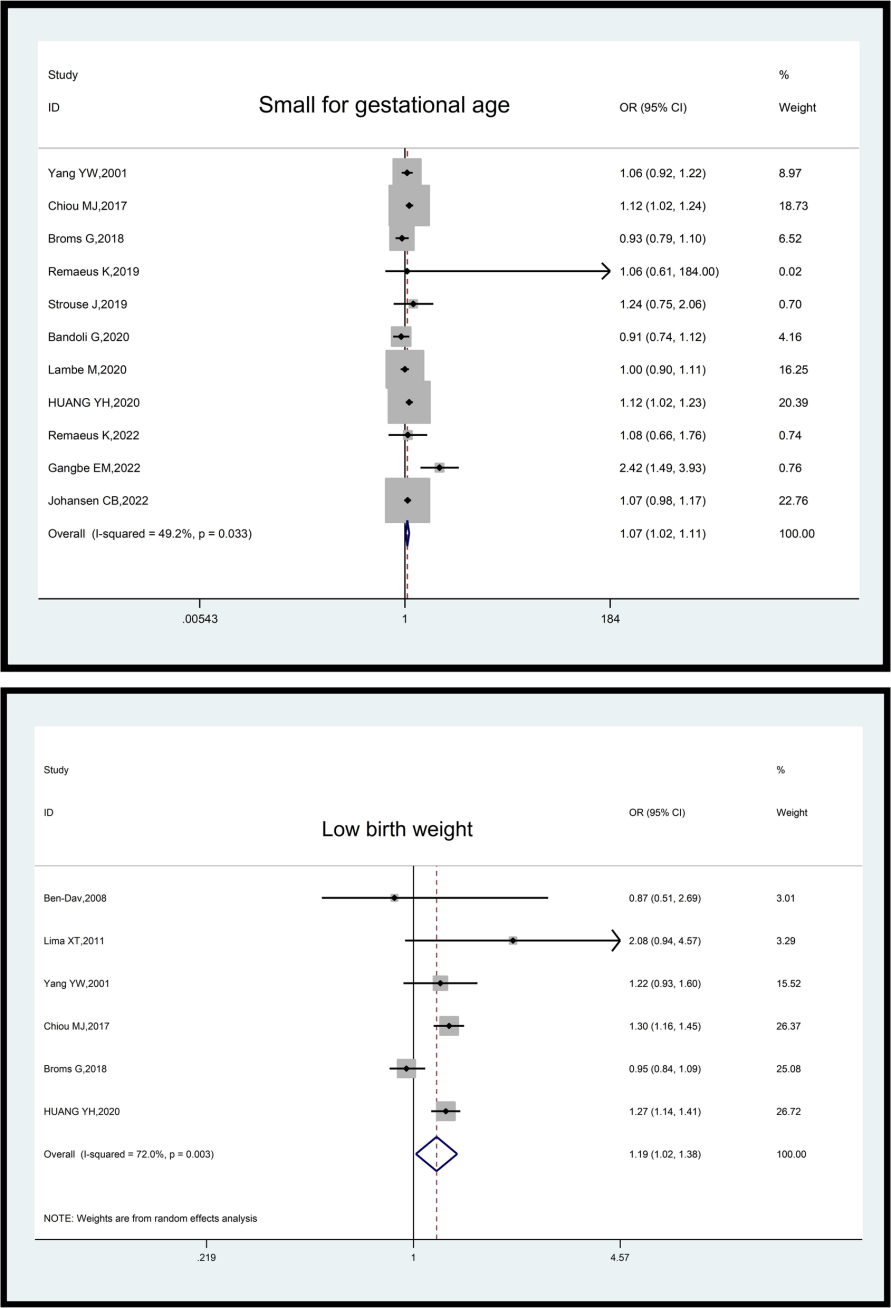
Impact of psoriasis on neonatal outcomes (Small for gestational age and Low birth weight)

The analysis of 12 studies involving a total sample size of 23,110,835 pregnant women (71,168 cases and 23,039,667 controls) revealed that psoriasis was associated with an increased odds of small for gestational age (OR: 1.07, 95% CI: 1.02–1.11, *p*-value = 0.05). The included studies exhibited moderate heterogeneity (I^2^ = 49.2%; *p*-value = 0.033) (Fig. 5).

In the analysis of six studies comprising 5,656,189 pregnant women (17,610 cases and 5,638,579 controls), it was found that psoriasis increased the odds of low birth weight (OR: 1.19, 95% CI: 1.02–1.38, *p*-value = 0.001). The included studies demonstrated moderate heterogeneity (I^2^ = 72%; *p*-value = 0.003) (Fig. 5).

**Fig. 6:**
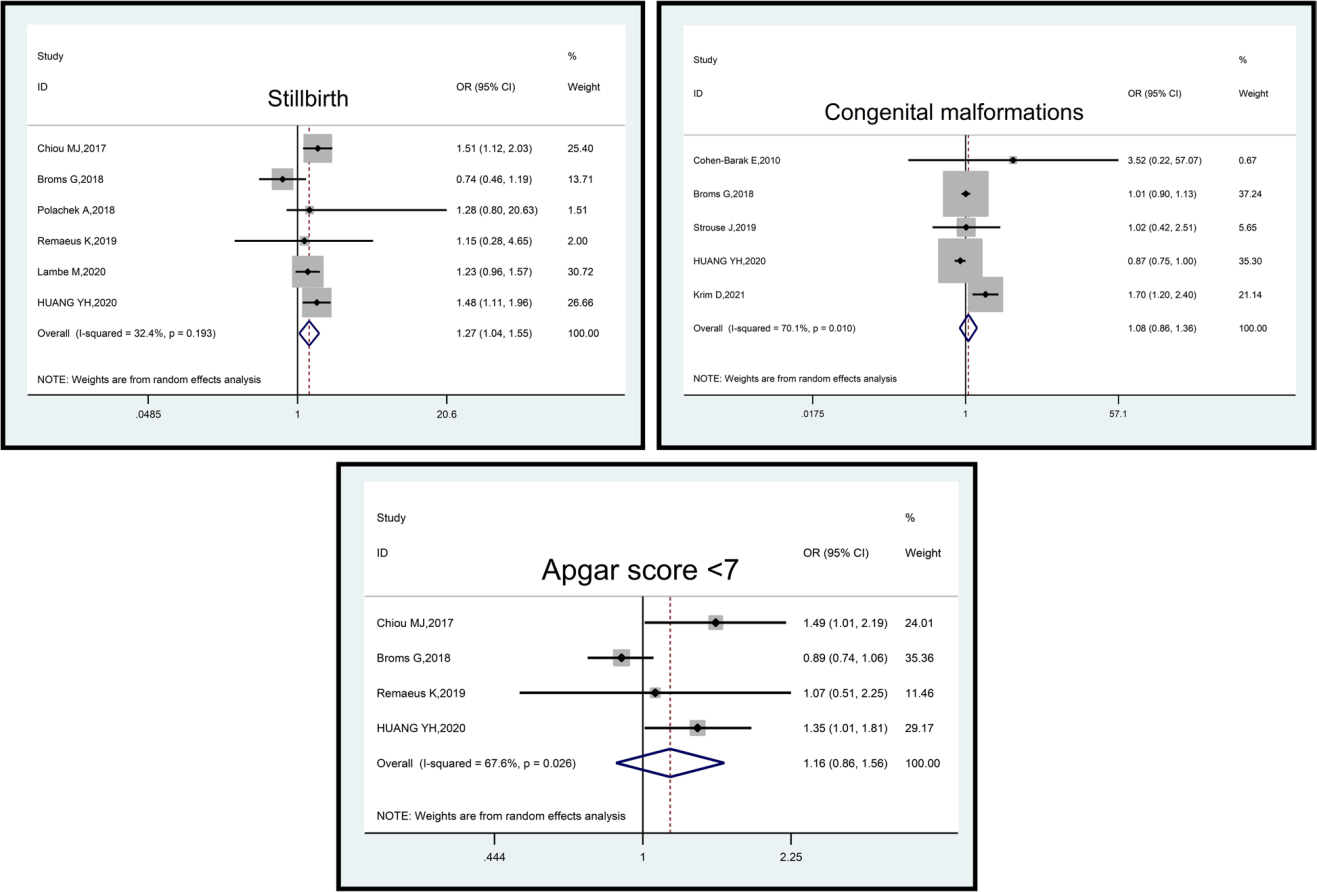
Impact of psoriasis on neonatal outcomes (Stillbirth, Congenital malformations, and Apgar score < 7)

Among the six studies involving 7,362,801 pregnant women (49,927 cases and 7,312,874 controls), the analysis indicated that psoriasis was associated with an elevated odds of stillbirth OR:1.27, 95% CI: 1.04–1.55, *p*-value = 0.023). The heterogeneity among these studies was moderate (I^2^ = 32.4%; *p*-value = 0.193), which was not statistically significant (Fig. 6).

Analyzing five studies encompassing 20,256,126 pregnant women (26,902 cases and 20,229,224 controls), it was found that psoriasis did not have a significant association with congenital malformations (OR:1.08, 95% CI: 0.86–1.36, *p*-value = 0.514). The included studies exhibited moderate heterogeneity (I^2^ = 70.1%; *p*-value = 0.010) (Fig. 6).

Finally, in the analysis of four studies involving 5,695,070 pregnant women (16,365 cases and 5,678,705 controls), no significant association between psoriasis and Apgar score < 7 (OR: 1.16, 95% CI: 0.86–1.56, *p*-value = 0.322) was observed. The included studies demonstrated moderate heterogeneity (I^2^ = 67.6%; *p*-value = 0.026) (Fig. 6).

#### Subgroup analysis

To investigate the source of heterogeneity, we performed several subgroups analysis based on the continent type and study quality. The results showed that heterogeneity could be explained by the region of conducted the study and the quality of included studies (Table 2). The pooled OR for stillbirth (OR: 1.49, 95% CI: 1.22–1.84) in Asian countries, cesarean delivery (OR: 1.36, 95% CI: 1.03–1.79) in European countries, and gestational hypertension (OR: 1.58, 95% CI: 1.13–2.21) in the American countries was the highest. In addition, the odds for (pre)eclampsia (OR: 1.32, 95% CI: 1.17–1.50) in high-quality articles, and cesarean delivery (OR: 1.42, 95% CI: 1.06–1.90) in low-quality articles were the highest.

**Table 2:** Subgroup analysis of the impact of psoriasis on maternal and neonatal outcome based on odds ratio

**Variables**	**Subgroup**														
	**Continent type**									**Study quality**					
	**Asia**			**Europe**			**America**			**High**			**Low**		
	OR^11^(CI)^12^	I^2^ (%)	*p*-value^13^	OR(C)	I^2^ (%)	*p*-value	OR (CI)	I^2^ (%)	*p*-value	OR(CI)	I^2^ (%)	*p*-value	OR(CI)	I^2^ (%)	*p*-value
**LBW**^**1**^	1.28(1.18–1.37)	0.0	0.792	0.95(0.83–1.08)	-	-	2.08(0.94–4.59)	-	-	1.19(0.88–1.61)	86.7	0.001	1.26(1.14–1.39)	0.0	0.659
**PTB**^**2**^	1.10(0.99–1.23)	0.0	0.885	1.07 (1.1–1.13)	78.7	0.001	1.33(1.21–1.47)	63.4	0.042	1.12(1.05–1.19)	77.9	0.0001	1.13(1.06–1.19)	804	0.002
**CM**^**3**^	0.87(0.76–1.01)	0.0	0.325	1.01(0.90–1.13)	-	-	1.57(1.08–2.26)	-	-	1.23(0.81–1.85)	74.5	0.020	0.87(0.76–1.01)	-	-
**SGA**^**4**^	1.11(1.04–1.18)	0.0	0.778	1.02(0.96–1.09)	0.0	0.647	1.08(0.90–1.29)	85.2	0.001	1.08(1.02–1.15)	58.9	0.023	1.05(0.99–1.13)	33.4	0.212
**AS**^**5**^	1.40(1.11–1.77)	0.0	0.690	0.90(0.76–1.07)	-	0.636	-	-	-	1.10(0.75–1.60)	67.6	0.026	1.35(1.01–1.81)	-	-
**SB**^**6**^	1.49(1.22–1.84)	0.0	0.924	1.02(0.70–1.50)	42.3	0.177	1.28(0.25–6.50)	-	-	1.12(0.69–1.82)	51.8	0.101	1.33(1.11–1.60)	0.0	0.335
**PRE**^**7**^	1.44(1.17–1.78)	51.7	0.126	1.12(1.04–1.21)	0.0	0.637	1.36(1.22–1.52)	0.0	0.565	1.32(1.17–1.50)	45.7	0.117	1.24(1.01–1.53)	79.5	0.002
**CD**^**8**^	1.21(0.99–1.47)	80	0.0001	1.36(1.03–1.79)	87.2	0.001	1.25(1.02–1.54)	79.5	0.002	1.19(1.08–1.30)	75.8	000.1	1.42(1.06–1.90)	87.7	0.001
**GD**^**9**^	1.22(1–1.49)	55.4	0.134				1.28(1.16–1.41)	0.0	0.727	1.27(1.17–1.38)	0.0	0.797	1.20(1.04–1.39_)	48	0.125
**GH**^**10**^	1.42(1.12–1.80)	-	-	1.26(1.11–1.42)	25.8	0.260	1.58(1.13–2.21)	-	-	1.25(1.03–1.51)	28.2	0.248	1.38(1.22–1.56)	0.0	0.797

^1^Low birth weight

^2^Preterm birth

^3^Congenital malformations

^4^Small for gestational age

^5^Apgar score < 7

^6^Stillbirth

^7^(pre) eclampsia

^8^Cesarean delivery

^9^Gestational diabetes

^10^Gestational hypertension

^11^Odds ratio

^12^Confidence interval

^13^*p*-value for heterogeneity test

#### Publication bias

The Egger test did not show a publication bias in different outcomes except for the preterm birth outcome (*p*-value = 0.024).

## Sensitivity analysis

Excluding one study in the sensitivity analysis did not have a significant effect on the results in terms of pregnancy and neonatal outcomes.

## Discussion

To obtain a conclusion about the association between maternal psoriasis and the risk of adverse pregnancy as well as neonatal outcomes in pregnant women, we performed a systematic review and meta-analysis by compiling the available evidence. This study showed that psoriasis could increase the risk of some pregnancy-related indicators like gestational diabetes, cesarean delivery, (pre)eclampsia, gestational hypertension, preterm birth, spontaneous abortion, and also some neonatal-related outcomes like Low birth weight, small for gestational age, Apgar score < 7, and stillbirth.

Our findings indicated that psoriasis increased the risk of preterm birth, which was consistent with other studies conducted in the United States (2017), Taiwan (2020), Swedish (2019), and California (2019) [46–49]. However, these results were inconsistent with the study conducted in Denmark (2018) showing no significant relationship between psoriasis and preterm birth [13]. The diversity between studies could be explained by the different sample sizes. Compared to other studies, the sample size of a study conducted in Denmark included a lower sample size. The identification of increased risk of preterm birth is an important finding, as it plays a key role in neonatal morbidity (congenital malformations, Low birth weight) and mortality [50, 51]. Preterm birth is complex, and the underlying mechanisms, risk factors, and etiology are not fully understood. Premature birth is associated with increased morbidity and mortality both early and late in life [48, 52].

Our findings demonstrated that psoriasis increased the risk of cesarean delivery. This finding is consistent with other studies [9, 13]. However, our result is inconsistent with two studies conducted in Boston, USA (2012) and Taiwan (2011). In the study conducted in Boston, which included a retrospective cohort of 122 mothers with psoriasis, and the study conducted across Taiwan, which included 1,463 mothers with psoriasis, it was shown that there was no association between cesarean delivery and psoriasis [53, 54]. In the present study and other studies, the risk of cesarean delivery increased in women with psoriasis, which is an expected result because an increased risk of cesarean delivery for chronic inflammatory diseases has been described in the literature [13, 48, 55, 56].

The results showed that psoriasis increased the risk of gestational diabetes, (pre)eclampsia, gestational hypertension, and spontaneous abortion. These findings are in line with several studies [13, 47, 57]. Gestational diabetes and gestational hypertension were associated with the development of diabetes and cardiovascular diseases in later stages of life, showing the benefits of preventive measures in this group [58]. In our study, women with psoriasis had an increased risk of (pre)eclampsia, regardless of severity. Our finding regarding the increased risk of (pre)eclampsia in women with severe psoriasis is consistent with a previous population-based study in Taiwan. This study found a positive association between severe psoriasis and Pre-Eclampsia. However, mild psoriasis was not associated with (pre)eclampsia. Such differences may be explained by the relatively limited number of psoriasis patients in the previous study and the different definitions of psoriasis severity used [54].

We found that psoriasis had a significant impact on some neonatal outcomes like Low birth weight, small for gestational age, Apgar score < 7, and stillbirth. These findings are consistent with several studies conducted around the world [13, 49, 54]. A study conducted in Sweden showed that no increased risks were observed for stillbirth and an Apgar score < 7, which is inconsistent with the current study [48]. Available pieces of evidence suggest that increased immune activity in psoriasis could be related to a higher risk of giving birth to Low-birth -weight infants. In support of this finding, it should be noted that immune disorders, like rheumatoid arthritis and inflammatory bowel disease, are associated with pregnancy complications, including Low birth weight and preterm birth [54].

An increased risk of adverse pregnancy and neonatal outcomes associated with psoriasis has been cited in many sources [13, 46, 49, 52]. Quantifying additional risks and associated pathways provides insight into the underlying causes of adverse pregnancy and neonatal outcomes and can shape intervention strategies [59]. As the disease affects the risk of preterm delivery in affected women, special attention to pregnant women is warranted. Women with psoriasis should be monitored individually during pregnancy, and an intervention aimed at preventing pregnancy complications should be done. Clinically, these interventions can be implemented with counseling and prevention efforts, especially in the case of (pre)eclampsia or high blood pressure for women with psoriasis. Both dermatologists and gynecologists should be familiar with the potential consequences of pregnancy in women with psoriasis and monitor their health during pregnancy as well as their infants. It may be an important goal, but with the emergence of new therapeutic options and the continuous collection of more data for their safety during pregnancy, it has become more realistic and important [13, 47, 48, 53, 54, 57].

This study has four limitations. First, due to some biases (selection, or information biases) in the observational studies, the interpretation of evidence from observational studies requires caution. Therefore, causal links of risk estimates cannot be determined based on the nature of observational studies. Second, although the quality of included studies was generally high, not all studies were adequately adjusted for all potential confounders. We could not fully control the confounding factors either. This may lead to underestimation or overestimation of the risk estimates for the presence of psoriasis diseases. Third, various sources of heterogeneity existed in the included studies possibly due to the varied study designs, data materials, analytical approaches, periods covered, as well as the quality of the studies. Fourth, we had to omit several studies due to different definitions of psoriasis, or inadequate presented information. It is important to mention that the calculated measures could change if we had included the removed studies.

## Conclusion

The present study provides strong evidence of the association between psoriasis and pregnancy and neonatal outcomes. Our results strengthen the importance of close monitoring of the mothers’ clinical status before and during pregnancy. Both dermatologists and gynecologists should be familiar with the potential consequences of pregnancy in women with psoriasis and monitor their health during pregnancy as well as their infants.

### Supplementary Information

**Additional file 1.**

**Additional file 2.**

## Data Availability

Data are available upon email to the correspondence author.
