# Supplementary material for: Impact of maternal psoriasis on adverse maternal and neonatal outcomes: a systematic review and meta-analysis
Source: BMC Pregnancy Childbirth. 2023 Sep 30;23:703. doi: 10.1186/s12884-023-06006-5 (PMC10543305; doi:10.1186/s12884-023-06006-5)
Supplement: Supplementary file 1 — Additional file 1. [file 12884_2023_6006_MOESM1_ESM.docx]

**Additional file 1**

**Search strategy**

**PubMed**

#1 psoriasis [MeSH Terms]

#2 psoriasis [Title/Abstract]

#3psoriasis*[Title/Abstract]

#4 (#1 OR #2 OR #3)

#5 Arthritis, Psoriatic [MeSH Terms]

#6 "Arthritis, Psoriatic"[Title/Abstract]

#7 Arthritis, Psoriatic*[Title/Abstract]

#8 (#5 OR #6 OR #7)

#9 (#4 OR #8)

#10 "maternal outcome"[Title/Abstract]

#11 maternal outcome*[Title/Abstract]

#12 (#10 OR #11)

#13 Cesarean*[Title/Abstract])

#14 Cesarean Sections*[Title/Abstract]

#15 "Cesarean Sections"[Title/Abstract]

#16 Caesarean operation*[Title/Abstract]

#17 Cesarian*[Title/Abstract]

#18 Cesarean Section [MeSH Terms]

#19 C-Section*[Title/Abstract]

#20 (#13 OR #14 OR #15 OR #16 OR #17 OR #18 OR #19)

#21 ((Abortion, Spontaneous [MeSH Terms])

#22 ("Abortion, Spontaneous"[Title/Abstract]))

#23 (Abortion, Spontaneous*[Title/Abstract])

#24 (#21 OR #22 OR #23)

#25 (pre) eclampsia [Title/Abstract]

#26 Pre-Eclampsia*[Title/Abstract]

#27 (pre) eclampsia [MeSH Terms]

#28 (#25 OR #26 OR #27)

#29 Eclampsia [MeSH Terms]

#30 Eclampsia*[Title/Abstract]

#31 (#29 OR #30)

#32 "Hypertension, Pregnancy-Induced"[Title/Abstract]

#33 Hypertension, Pregnancy-Induced [MeSH Terms]

#34 (#32 OR #33)

#35 "Diabetes, Gestational"[Title/Abstract]

# 36 Diabetes, Gestational [MeSH Terms]

#37 (#35 OR #36)

#38 Postpartum Hemorrhage [MeSH Terms]

#39 "Postpartum Hemorrhage"[Title/Abstract]

# 40 (#38 OR #39)

# 41 Anemia [MeSH Terms]

# 42 Anemia*[Title/Abstract]

#43(#41 OR #42)

#44 (#12 OR#20 OR#24 OR#28 OR#31 OR#34 OR#37 OR# 40 OR#43)

#45 neonatal outcome*[Title/Abstract]

# 46"neonatal outcome"[Title/Abstract]

#47(#45 OR #46)

#48 Infant, Low Birth Weight [MeSH Terms]

#49 (infant low birth weight*[Title/Abstract]

#50 "Infant, Low Birth Weight"[Title/Abstract]

#51(48 OR #49 OR 50)

#52 Fetal Macrosomia [MeSH Terms]

#53"Fetal Macrosomia"[Title/Abstract]

#54 Fetal Macrosomia*[Title/Abstract]

#55 (52 OR #52 OR 54)

#56 Myocardial Infarction[MeSH Terms])

# 57 "Myocardial Infarction"[Title/Abstract]

#58 Myocardial Infarction*[Title/Abstract]

#59 (56 OR #57 OR 58)

# 60 Infant, Small for Gestational Age [MeSH Terms]

#61"Infant, Small for Gestational Age"[Title/Abstract]

# 62 (60 OR #61)

#63 Infant, Premature [MeSH Terms]

#64 "Infant, Premature"[Title/Abstract]

# 65 (63 OR # 64)

# 66 Stillbirth [MeSH Terms]

# 67 Stillbirth*[Title/Abstract]

# 68 (66 OR # 67)

#69 Congenital Abnormalities [MeSH Terms]

#70 "Congenital Abnormalities"[Title/Abstract]

#71 (69 OR # 70)

#72 "intra-uterine growth restriction"[Title/Abstract]

# 73 intra-uterine growth restriction*[Title/Abstract]

#74 (72 OR # 73)

# 75 Apgar score [MeSH Terms]

#76 "Apgar score"[Title/Abstract])

# 77 (75 OR # 76)

#78(# 47 OR # 51 OR # 55 OR # 59 OR # 62 OR # 65 OR # 68 OR # 71 OR # 74 OR # 77 )

# 79 (#44 OR #78)

#80 (#9 AND #79) **= 1414**
