# Supplementary material for: Impact of maternal psoriasis on adverse maternal and neonatal outcomes: a systematic review and meta-analysis
Source: BMC Pregnancy Childbirth. 2023 Sep 30;23:703. doi: 10.1186/s12884-023-06006-5 (PMC10543305; doi:10.1186/s12884-023-06006-5)
Supplement: Supplementary file 2 — Additional file 2. [file 12884_2023_6006_MOESM2_ESM.docx]

**Additional file 2**

**Effect size based on RR**

**The impact of psoriasis on maternal outcomes**

Analysis of three studies, with a sample size of 4,427 pregnant women (627 cases and 3,800 controls), showed that psoriasis increased the risk of preterm birth. RR = 1.82 (95% CI: 1.37-2.41, p-value = 0.002).

Analysis of three studies, with a sample size of 4,427 pregnant women (627 cases and 3,800 controls), showed that psoriasis increased the risk of (pre) eclampsia. RR = 1.61 (95% CI: 1.12-2.32, p-value = 0.001).

Analysis of two studies, with a sample size of 1,393 pregnant women (297 cases and 1,096 controls), showed that psoriasis increased the risk of caesarian delivery. RR = 1.52 (95% CI: 1.27-1.82, p-value = 0.002).

**Publication bias**

The result of the eager test (Preterm birth: p-value =0.602, Pre-Eclampsia: p-value = 0.117, and caesarian delivery: p-value = 0.317), indicated a lack of publication bias.

**Sensitivity analysis**

The omission of one study in the sensitivity analysis did not have a significant effect on the pregnancy outcomes.
